# A novel Alu-based real-time PCR method for the quantitative detection of plasma circulating cell-free DNA: Sensitivity and specificity for the diagnosis of myocardial infarction

**DOI:** 10.3892/ijmm.2014.1991

**Published:** 2014-11-05

**Authors:** XIAOLI LOU, YANQIANG HOU, DONGYU LIANG, LIANG PENG, HONGWEI CHEN, SHANYUAN MA, LURONG ZHANG

**Affiliations:** 1 Department of Central Laboratory, Songjiang Hospital Affiliated First People’s Hospital, Shanghai Jiao Tong University, Shanghai 201600, P.R. China; 2 Department of Radiation Oncology, Shands Cancer Center, University of Florida, Gainesville, FL 32610, USA

**Keywords:** cell-free DNA, Alu, real-time PCR, myocardial infarction

## Abstract

In the present study, we aimed to develop and validate a rapid and sensitive, Alu-based real-time PCR method for the detection of circulating cell-free DNA (cfDNA). This method targeted repetitive elements of the Alu reduplicative elements in the human genome, followed by signal amplification using fluorescence quantification. Standard Alu-puc57 vectors were constructed and 5 pairs of specific primers were designed. Valuation was conducted concerning linearity, variation and recovery. We found 5 linear responses (R_1–5_=0.998–0.999). The average intra- and inter-assay coefficients of variance were 12.98 and 10.75%, respectively. The recovery was 82.33–114.01%, with a mean recovery index of 101.26%. This Alu-based assay was reliable, accurate and sensitive for the quantitative detection of cfDNA. Plasma from normal controls and patients with myocardial infarction (MI) were analyzed, and the baseline levels of cfDNA were higher in the MI group. The area under the receiver operating characteristic (ROC) curve for Alu1, Alu2, Alu3, Alu4, Alu5 and Alu (Alu1 + Alu2 + Alu3 + Alu4 + Alu5) was 0.887, 0.758, 0.857, 0.940, 0.968 and 0.933, respectively. The optimal cut-off value for Alu1, Alu2, Alu3, Alu4, Alu5 and Alu to predict MI was 3.71, 1.93, 0.22, 3.73, 6.13 and 6.40 log copies/ml. We demonstrate that this new method is a reliable, accurate and sensitive method for the quantitative detection of cfDNA and that it is useful for studying the regulation of cfDNA in certain pathological conditions. Alu4, Alu5 and Alu showed better sensitivity and specificity for the diagnosis of MI compared with cardiac troponin I (cTnI), creatine kinase MB (CK-MB) isoenzyme and lactate dehydrogenase (LDH). Alu5 had the best prognostic ability.

## Introduction

Circulating cell-free DNA (cfDNA) mainly originates from programmed cell death or acute cellular injury and reflects the extent of cellular damage. cfDNA can be determined in the serum or plasma from healthy individuals at low concentrations, ranging from 2.5 to 27.0 ng/ml (1). High concentrations of cfDNA have been widely described in a variety of clinicopathological conditions, such as malignancies, trauma, infection, pregnancy-associated disorders and autoimmune diseases (2–5). It has been reported that the levels of cfDNA in serum and plasma are significantly increased in patients with colorectal, ovarian and testicular cancer (6–8).

Alu repeats, 300 nucleotides long with a copy number of approximately 1.4×10^6^ per genome, are short interspersed elements (SINEs) and are the most abundant sequences in the human genome. Alu sequences account for >10% of the genome and are abundant in blood (9–11). Therefore, Alu-based real-time PCR is a potentially sensitive approach for the measurement of human cfDNA in blood. In the present study, we designed 5 different sizes of Alu primers (201, 170, 147, 113 and 76 bp) and used real-time PCR for the measurement of cfDNA. We aimed to determine whether Alu-based real-time PCR can serve as an effective tool for the detection of cfDNA.

Myocardial infarction (MI) is the end result of coronary artery disease and a condition associated with the apoptosis and death of cardiomyocytes. cfDNA is mainly released following programmed cell death or acute cellular injury; therefore, we detected the cfDNA in plasma from patients with MI plasma using Alu-based real-time PCR.

## Materials and methods

### Standard Alu-puc57 vector construction and verification

Alu-puc57 vectors were constructed by cloning the entire Alu sequence amplified by PCR into the puc57 vector (Sangon Biotech Co., Ltd., Shanghai, China) at the *Eco*RI and *Hin*dIII restriction sites. The Alu sequence was amplified using a forward primer with an *Eco*RI restriction site (5′-gaa ttc aga cca tcc tgg cta aca cg-3′) and a reverse primer with a *Hin*dIII restriction site (5′-aag ctt aga cgg agt ctc gct ctg tc-3′). PCR was performed at 95°C for 2 min, 94°C for 20 sec, 55°C for 30 sec, and 72°C for 30 sec, repeated for 35 cycles. Extension was performed at 72°C for 10 min and 4°C for 10 min. The reconstructed plasmid, Alu-puc57, was verified by PCR sequencing.

### Sample preparation and clinical information

We examined 120 patients diagnosed with MI (40–80 years of age; 60 males and 60 females) at the Songjiang Hospital Affiliated to the First People’s Hospital, Shanghai Jiao Tong University, Shanghai, China. Peripheral blood samples were collected within 6 h of admission after the onset of chest pain. The patients had been diagnosed with MI on the basis of clinical symptoms, 12-lead electrocardiography and positive cardiac biomarkers. A two-site immunoenzymometric assay was used to test for cardiac troponin I (cTnI), creatine kinase (CK) MB (CK-MB) isoenzyme, myoglobin (MYO) and lactate dehydrogenase (LDH) with the Cobas c501 System analyzer (Hoffmann-La Roche, Basel, Switzerland). Blood samples were collected from 60 healthy control subjects (40–80 years of age; 30 males and 30 females). These individuals had no history of autoimmune disease, tissue injury, or trauma at the time of examination and their hematological-biochemical profile was normal. All samples were anonymous and the Ethics Committee of the Shanghai Songjiang District Central Hospital approved this study. Participants provided their written informed consent to participate in this study and the ethics committees approved this consent procedure. Fresh blood was collected into tubes containing clot activation additive for serum and into tubes containing EDTA for plasma (Shanghai Kehua Bio-Engineering Co., Ltd., Shanghai, China). Whole blood was separated at 4°C by centrifugation at 1,600 × g for 10 min. Plasma samples were stored at −80°C. After collection, all samples were analyzed for cfDNA on the same day in order to minimize testing error.

### Primer design

**Figure 1:**
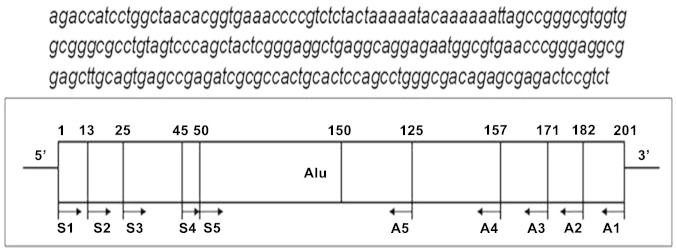
Sequence of Alu and scheme for amplification. PCR primers are shown as lines with arrowheads to indicate 5′→3′ orientation relative to the Alu sequence. The sequences of each primer are shown in Table I.

Five primer pairs amplifying different fragments of Alu were designed to quantify the amount of cfDNA and to characterize the fragmentation pattern. The Alu1, Alu2, Alu3, Alu4 and Alu5 sequences are shown in Table I. The sequences of Alu and the primer model are shown in Fig. 1. The products of the 5 primers were 201, 170, 147, 113 and 76 bp, respectively.

### Detection of cfDNA in plasma by real-time PCR

Real-time PCR was performed in triplicate on an ABI 7500 (Applied Biosystems, Foster City, CA, USA). Each 50 μl PCR reaction consisted of 25 μl SYBR-Green mix (Takara Bio, Dalian, China), 2 μl forward/reverse primer (10 μM) and 5 μl plasma sample. The cycling conditions were 90°C for 30 sec, followed by 40 cycles at 95°C for 5 sec and 60°C for 34 sec. The specificity of the PCR products was confirmed by melting curve analysis. The running time required 33 min for the cfDNA assay. Each run included water blanks and serial dilutions of an external standard, Alu-puc57 vector. The resultant copies of different primers were calculated into log values. The copies of Alu1, Alu2, Alu3, Alu4 and Alu5 were added and calculated by logarithmic conversion and recorded as Alu addition.

### Linearity, lower limit of detection and variation in the Alu-based real-time PCR

The samples used for the evaluation of the method were dilutions of the standard Alu-puc57 vector with the original content of 1×10^10^ copies/ml. The linearity of cfDNA quantification was assessed using serial dilutions 1×10^6^, 1×10^7^, 1×10^8^, 1×10^9^ and 1×10^10^ copies/ml of the standard Alu-puc57 vector. The samples were tested in triplicate in 3 different runs. The dilution concentrations for the variation analysis were 4×10^6^, 8×10^7^ and 2×10^9^ copies/ml. The intra-sample reproducibility of Alu-based real-time PCR was evaluated with samples tested 13 times in the same batch. The inter-sample reproducibility was evaluated with samples tested in each run for 20 consecutive days.

The lower limit of detection was used to demonstrate the sensitivity of detection of Alu-based real-time PCR, which was defined as the mean ± 2 SDs of 0 copies/ml cfDNA.

### DNA recovery by real-time PCR

The reliability of Alu-based real-time PCR was examined by measuring the recovery of different Alu sequences from spiked plasma samples. The DNA concentrations in each of 2 randomly selected standard samples were first measured by real-time PCR. Analytical recovery was evaluated by the addition of 0.1 ml of the calibrator at concentrations of 1.2×10^7^ or 1×10^10^ copies/ml to 0.9 ml of 4×10^6^ or 2×10^9^ copies/ml sample, respectively. Five microliters of each mixture were subsequently loaded into separate detection wells in triplicate for DNA quantification by real-time PCR. Recovery was calculated using the following equation:

$$\text{Recovery}=\frac{\text{Measurement by real}-\text{time PCR}}{\text{Baseline}+\text{spiked}}\times 100$$

where recovery is the total amount of DNA measured by real-time PCR in the spiked sample (measured by real-time PCR), over the sum (expected) of the DNA quantity in samples before spiking (baseline) and the amount of DNA spiked (spiked), expressed as a percentage.

### Statistical analysis

Quantitative data are expressed as the means ± SD. Statistical analysis was carried out using SPSS software for Windows version 13.0. Where appropriate, ANOVA was first used to ascertain whether there was a significant difference between the means. In order to evaluate the predictive value of Alu-based real-time PCR, receiver operating characteristic (ROC) curve analysis was applied and the area under the curve (AUC) was compared. All statistical tests were two-sided, and the significance level was set at p<0.05.

## Results

### Verification of Alu-p57 vector and development of real-time PCR

**Figure 2:**
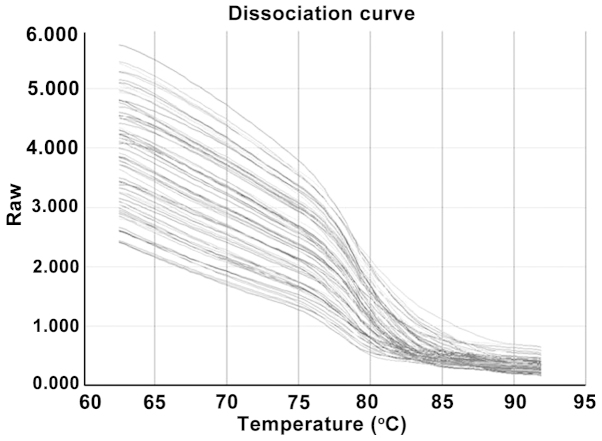
Melting curve of amplification from each of the standard dilutions and plasma samples.

**Figure 3:**
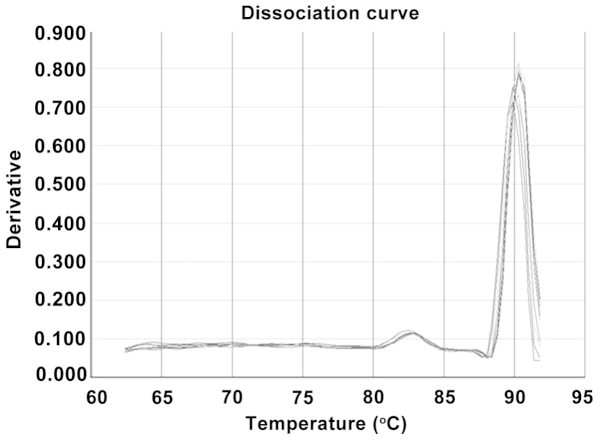
Melting peak transformed from the melting curve.

The reconstructed plasmid Alu-puc57 was verified by PCR sequencing which confirmed the accurate bases (data not shown). Five pairs of primers that framed the human Alu sequence were selected and constructed to detect quantitative changes in cfDNA in human peripheral blood (Fig. 1). It was possible to amplify 5 DNA sequences of Alu. The primers are shown in Table I. Correspondingly, the products generated by real-time PCR exhibited a range of sizes at 201, 170, 147, 113 and 76 bp. When the different standard dilutions and plasma samples were melted during the amplification process, a sharp composite melting curve was obtained (Fig. 2). As each of the reactions was heated toward the denaturation temperature, the fluorescence levels decreased to background levels within a 7°C range between 80 and 87°C. Following background subtraction, the derivative of the curve was used to convert the melting curves to a melting peak (Fig. 3). Multiple single peaks occurring in a narrow range of melting temperature indicate that the method was target specific.

### Linearity

**Figure 4:**
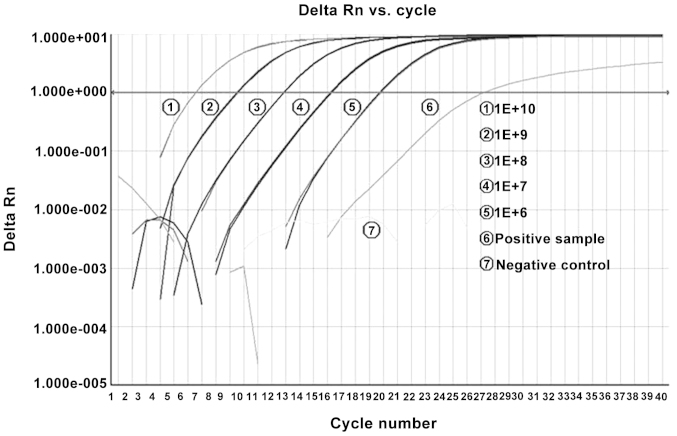
Amplification plots of real-time PCR for the Alu sequence. The x-axis is the cycle number of real-time PCR. The y-axis denotes the fluorescence intensity above background. Each dilution was assayed in duplicate.

**Figure 5:**
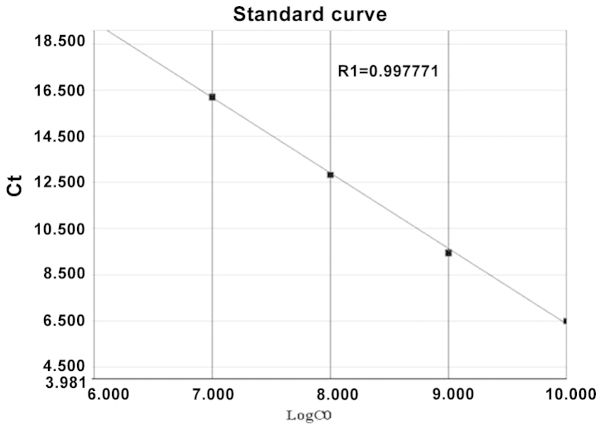
Linearity of Alu-based real-time PCR. The y-axis is the threshold cycle (CT) and the x-axis denotes the log of the starting quantity of template. Each dilution was measured in duplicate. Evaluation of the linearity found 5 linear responses (R_1_=0.998, R_2_=0.998, R_3_=0.998, R_4_=0.998, R_5_=0.999).

We performed experiments using several 4-fold dilutions starting from 1×10^10^ copies/reaction and ending at 1×10^6^ copies/reaction, resulting in evenly spaced amplification curves (Fig. 4). The evaluation of the Alu-based real-time PCR assay, shown with serial 10-fold dilutions of the standard Alu-puc57 plasmid, found 5 linear responses (R_1_=0.998, R_2_=0.998, R_3_=0.998, R_4_=0.998 and R_5_=0.999) (Fig. 5).

### Lower limit of detection

The lower limits of Alu1, Alu2, Alu3, Alu4 and Alu5 for detection with Alu-based real-time PCR, which were defined as the means ± 2 SD of 15 replicates, the zero standard, were 8.89, 6.98, 7.08, 10.12 and 9.56 copies/ml, respectively.

### Variation

For analysis of the variation of the Alu-based real-time PCR assay, we used 3 concentrations (2×10^4^, 4×10^5^ and 1×10^7^ copies/reaction) of diluted standard Alu-puc57 plasmids. The results for within-run reproducibility are presented in Table II, obtained by assaying 13 replicates of each sample in a single assay. Similarly, the results of between-run reproducibility are presented in Table III, for each concentration measured for 20 consecutive days.

### Recovery

As shown in Table IV, recovery was found to range from 82.33 to 114.01% with a mean recovery index of 101.26%. The recoveries corresponding to the 2 concentrations for each primer did not differ greatly, indicating that Alu-based real-time PCR was satisfactorily reliable and consistent when used to directly estimate DNA quantity in routine plasma samples.

### Analysis of cfDNA in clinical plasma samples from patients with MI and normal controls

**Figure 6:**
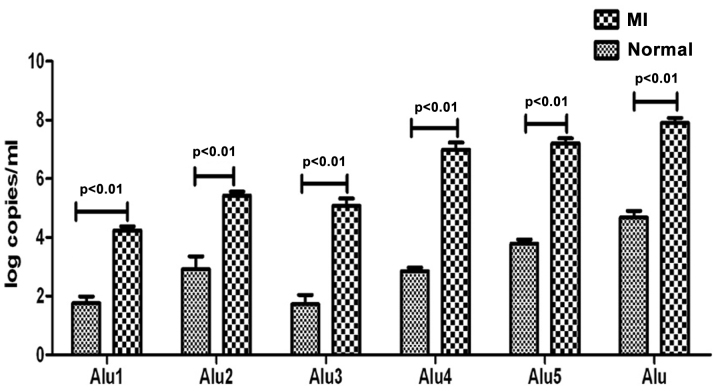
Analysis of cell-free DNA (cfDNA) in clinical plasma samples from normal controls and patients with myocardial infarction (MI). Patient plasma samples contained a higher concentration of cfDNA than the plasma samples from the normal controls (p<0.01).

The Alu-based real-time PCR assay was used to measure the levels of cfDNA in 120 plasma samples from patients with MI and 60 plasma samples collected from normal blood donors. The logarithmic mean value of the Alu1, Alu2, Alu3, Alu4, Alu5 and Alu cfDNA concentration was 1.75±0.24, 2.92±0.44, 1.72±0.32, 2.85±0.12, 3.79±0.14 and 4.67±0.24 log copies/ml in the plasma samples from the normal controls. By contrast, the MI plasma samples had a high concentration of cfDNA (4.24±0.16, 5.42±0.14, 5.08±0.25, 6.99±0.24, 7.21±0.17 and 7.90±0.17 log copies/ml). This indicated that the plasma samples from the patients with MI contained a higher concentration of cfDNA than the plasma samples from the normal controls (p<0.01) (Fig. 6).

### Diagnostic ability of Alu based real-time PCR for the detection of MI

**Figure 7:**
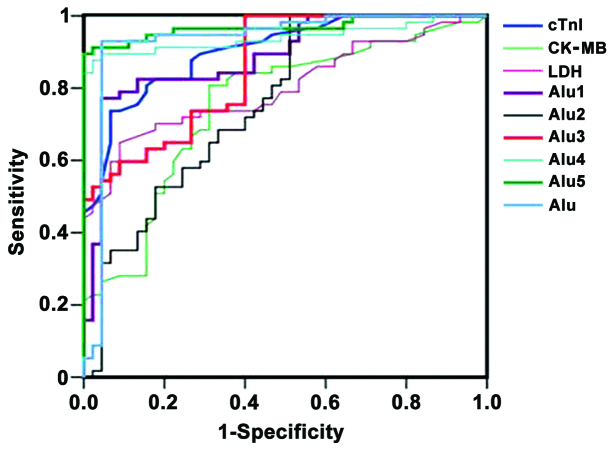
Receiver operating characteristic (ROC) curves of cardiac troponin I (cTnI), CK, creatine kinase MB (CK-MB) isoenzyme, lactate dehydrogenase (LDH), Alu1, Alu2, Alu3, Alu4, Alu5 and Alu. Alu4, Alu5 and Alu showed better prognostic ability for the diagnosis of myocardial infarction (MI).

To evaluate the diagnostic ability of Alu-based real-time PCR for discriminating MI patients from normal controls, the ROC curves of cTnI, CK, CK-MB, LDH, Alu1, Alu2, Alu3, Alu4, Alu5 and Alu were drawn (Fig. 7). The AUC for Alu1 was 0.887 [95% confidence interval (CI), 0.822–0.953], 0.758 (95% CI, 0.662–0.854) for Alu2, 0.857 (95% CI, 0.787–0.927) for Alu 3, 0.940 (95% CI, 0.889–0.990) for Alu 4, 0.968 (95% CI, 0.934–1.002) for Alu 5 and 0.933 (95% CI, 0.871–0.994) for Alu (Table V). The optimal cut-off value for Alu1 to predict MI was 3.71 log copies/ml (77.20% sensitivity and 95.60% specificity), for Alu2 1.93 log copies/ml (100% sensitivity and 48.95% specificity), for Alu3 0.22 log copies/ml (100% sensitivity and 60.0% specificity), for Alu4 3.73 log copies/ml (86.8% sensitivity and 95.6% specificity), for Alu5 6.13 log copies/ml (86.8% sensitivity and 100% specificity) and for Alu 6.40 log copies/ml (92.1 and 95.6% sensitivity) (Table V).

## Discussion

Our study demonstrates that this Alu-based real-time PCR method is a reliable, accurate and sensitive method for the quantitative detection of plasma cfDNA in patients with MI for the following reasons. Firstly, we constructed the standard Alu-puc57 vectors, designed 5 specific primers based on Alu sequences, and evaluated the linearity of this real-time PCR, which showed 5 good linear responses. Secondly, the variation and recovery evaluation indicated that this Alu-based assay was an accurate method. Finally, using ROC curve analysis to determine the performance of cfDNA as a diagnostic test for MI detection, the sensitivity and specificity of Alu4, Alu5 and Alu exceeded 85%, suggesting that they are adequate for MI screening, particularly Alu5.

The history of cfDNA dates back to 1948, when it was first reported in human blood (12). Higher concentrations of cfDNA have been reported in a variety of conditions associated with cell damage or apoptosis, such as cancer (13), trauma (14), pre-eclampsia (15), certain systemic autoimmune diseases (16), aging (17,18), prenatal screening (19,20), infection (21), leukemia (22) and sepsis (23–25). It has been reported that patients with systemic sclerosis with active disease present with significantly higher cfDNA concentrations than those with inactive disease (26). Higher cfDNA levels have been found in patients with breast cancer and systemic lupus erythematosus and rheumatoid arthritis (27–30). Increased cfDNA was reported for the first time in patients with MI that could complement troponin and CK-MB in a multiple marker test format in 2003 (31). Therefore, cfDNA levels may serve as a biomarker for the diagnosis and prognosis of different pathological diseases.

Alu repeats, 300 nucleotides long with a copy number of approximately 1.4×10^6^ per genome, are SINEs and are the most abundant sequences in the human genome. Alu sequences account for >10% of the genome and are abundant in blood (9–11). According to a high-throughput sequencing analysis performed in healthy individuals, the plasma cfDNA sequence representation mirrors that of the genome (32). Alu-based real-time PCR may be a potentially sensitive approach for the measurement of human cfDNA in blood.

In our study, we designed 5 primer pairs amplifying different fragments of Alu to quantify the amount of cfDNA and characterize the fragmentation pattern. The products of the 5 primers were 201, 170, 147, 113 and 76 bp. We set up a novel real-time PCR based on the Alu sequences. We designed 5 pairs of specific primers for different PCR products of Alu sequences. The melting curves were measured and multiple single peaks occurring in a narrow range of melting temperature indicated that the method was target-specific. Valuation was conducted concerning linearity, variation and recovery. Evaluation of the linearity found 5 linear responses. The average intra- and inter-assay coefficients of variance were 12.98 and 10.75%, respectively. The recovery was found to range from 82.33 to 114.01%, with a mean recovery index of 101.26%. Thus, this Alu-based assay is a reliable, accurate and sensitive method for the quantitative detection cfDNA.

The traditional spectrophotometric technique for the determination of the concentration of DNA is through the measurement of absorbance at a wavelength of 260 nm (33). The major disadvantages of this technique are the interference caused by contaminants in the process of nucleic acid preparation. Currently, several techniques, including PCR-based assays, fluorimetric methods and hybridization methods, have been used for the detection of cfDNA. PCR-based assays, including real-time PCR (34–37), duplex real-time PCR and methylation-specific PCR (38), are the most common methods available for the detection of cfDNA (39). PCR methods are based on the use of commercial DNA extraction kits for the purification of cfDNA. The use of gene-specific primers or probe sequences requires the optimization of PCR conditions. It has been shown that DNA quantification using fluorescent dyes is a simple method that can detect nearly all DNA fragments not specific for a DNA gene (40). Sang *et al* developed a method for the quantification of cfDNA by capillary zone electrophoresis with laser-induced fluorescence detection (41). Jing *et al* described a branched DNA (bDNA) method based on SINEs (Alu repeats) for the direct quantification of cfDNA in serum/plasma (42).

The present study builds on these previous methods; however, our approach and findings differ in several important ways. Firstly, as a result of the diversity of protocols, reagents and devices preventing a meaningful comparison of data from different laboratories, there is a need for the standardization of a highly sensitive molecular biology technique for the measurement of cfDNA. We constructed a standard Alu-puc57 vector that can be used as a standard substance or as a quality control in different laboratories. Secondly, we used real-time PCR to amplify the Alu sequence, which was more cost-effective and sensitive than the bDNA assay. Thirdly, we amplified the Alu sequences, which are specific genome repeats. However, other studies evaluate fragmentation of the whole genome. Other techniques measure the released mtDNA. These techniques bring us closer to producing reliable and quantitative results.

MI is the end result of coronary artery disease and the mortality rate associated with this disease worldwide has decreased from 10.5% in 1999 to 7.8% in 2008. The decrease is possibly explained by timely diagnosis that allows clinicians to adopt appropriate treatment (43). cTnI, CK, CK-MB and LDH have been used for the diagnosis of MI and for the follow-up of patients (44). Increased cfDNA in patients with MI may complement cTnI, CK-MB and MYO in a multiple marker format. Jing *et al* found that cfDNA was significantly higher in patients with MI compared to the controls (42). Chang *et al* found that the average concentration of cfDNA in 55 patients with MI was >10-fold higher than that in 274 normal controls. There was no correlation between the concentration of cfDNA and CK-MB or cTnI (31). However, Cui *et al* found a trend towards a positive association between peak plasma cfDNA and established markers of necrosis, such as CK-MB and cTnI (45). Destouni *et al* found that the concentration of cfDNA was significantly higher in patients with MI than that in a healthy control group using real-time PCR of the β-globin gene during hospitalization (46). The concentration of cfDNA has potential clinical value in monitoring progress and judging the prognosis of patients with MI (47). Shimony *et al* described a novel rapid fluorometric assay, the fluorochrome SYBR-Gold, which does not require prior processing of samples, for the quantification of cfDNA and found that peak cfDNA levels were significantly higher in patients compared with controls (48). Cui *et al* explored a bDNA-based Alu assay and reported that patients with acute coronary syndrome (ACS) showed a significant increase in plasma cfDNA concentrations compared with the controls. Moreover, they found positive correlations between cfDNA and Gensini scoring and Global Registry of Acute Coronary Events (GRACE) scoring in ACS (45).

In this study, we applied Alu-based real-time PCR to detect cfDNA in plasma samples from patients with MI. We found that the 5 different Alu PCR products were much higher in the MI group than in the normal individuals, which was consistent with the results of previous studies (31,42). We evaluated the prognostic ability of Alu-based real-time PCR for discriminating patients with MI from healthy controls, and ROC curves of cTnI, CK, CK-MB, LDH, Alu1, Alu2, Alu3, Alu4, Alu5 and Alu were drawn. The AUC for Alu1, Alu2, Alu3, Alu4, Alu5 and Alu was 0.887, 0.758, 0.857, 0.940, 0.968 and 0.933, respectively. In our study, Alu4, Alu5 and Alu showed better sensitivity and specificity for the diagnosis of patients with MI compared with cTnI, CK, CKMB and LDH. In particular, Alu5 displayed the best prognostic ability.

In conclusion, we developed a novel real-time PCR method based on Alu reduplicative sequences and proved that this is a reliable, accurate and sensitive method for the quantitative detection of cfDNA. This method may prove useful in studying cfDNA regulation in a variety of human pathological conditions.

## Figures and Tables

**Table I:** Primers of Alu sequences.

Primer name	Sequence	Tm (°C)	Size (bp)
S1	aga cca tcc tgg cta aca cg	56.8	200
A1	aga cgg agt ctc gct ctg tc	57.2	
S2	cta aca cgg tga aac ccc g	56.0	170
A2	cgc cca ggc tgg agt g	59.4	
S3	gtg aaa ccc cgt ctc tac taa aaa	54.7	147
A3	gag tgc agt ggc gcg at	58.8	
S4	tac aaa aaa tta gcc ggg cg	55.6	113
A4	gat ctc ggc tca ctg caa g	55.8	
S5	aaa att agc cgg gcg tg	53.4	76
A5	gtt cac gcc att ctc ctg c	56.6	

Tm, temperature.

**Table II:** Intra-assay variation.

Standard Alu DNA (copies/reaction)	Alu1 (n=13)		Alu2 (n=13)		Alu3 (n=13)		Alu4 (n=13)		Alu5 (n=13)	
				
	Mean ± SD	CV%	Mean ± SD	CV%	Mean ± SD	CV%	Mean ± SD	CV%	Mean ± SD	CV%
2.0×10^4^	2.51×10^4^±3196.791	12.70	2.62×10^4^±3504.8996	13.40	2.05×10^4^±2631.959	12.80	2.06×10^4^±3233.638	15.60	2.28×10^4^±3200	14.00
4.0×10^5^	4.23×10^5^±46278.38	10.90	4.63×10^5^±63388.274	13.70	4.79×10^5^±64519.7	13.50	4.04×10^5^±57378.77	14.20	4.39×10^5^±53333.94	12.10
1.0×10^7^	1.25×10^7^±1346627	10.80	1.19×10^7^±1239895.6	10.40	1.93×10^7^±2330,261	12.10	1.41×10^7^±2142322	15.20	1.28×10^7^±1692152	13.20

SD, standard deviation, CV, coefficient of variation.

**Table III:** Inter-assay variation.

Standard Alu DNA (copies/reaction)	Alu1 (n=20)		Alu2 (n=20)		Alu3 (n=20)		Alu4 (n=20)		Alu5 (n=20)	
				
	Mean ± SD	CV%	Mean ± SD	CV%	Mean ± SD	CV%	Mean ± SD	CV%	Mean ± SD	CV%
2.0×10^4^	2.77×10^4^±3647.336	13.20	2.55×10^4^±2533.836	9.94	2.47×10^4^±2299.805	9.31	2.41×10^4^±3286.043	13.60	2.98×10^4^±4288.356	14.40
4.0×10^5^	4.07×10^5^±36847.06	9.05	4.74Ex10^5^±58693.25	12.40	4.39×10^5^±54264.91	12.40	4.15×10^5^±56799.57	13.70	4.10×10^5^±33686.84	8.22
1.0×10^7^	1.26×10^7^±1133491	9.01	1.36×10^7^±1791659	13.10	1.46×10^7^±1926075	13.20	1.20×10^7^±1258028	10.40	1.17×10^7^±1586178	13.60

SD, standard deviation, CV, coefficient of variation.

**Table IV:** Recovery of Alu based real-time PCR assay.

Test set		Baseline (copies/reaction)	Spiked (copies/reaction)	Expected (copies/reaction)	Measured by real-time PCR (copies/reaction)	Recovery (%)
Alu1	Low range	2×10^4^	2×10^4^	4×10^4^	3.87×10^4^±6360	96.7±15.90
	High range	1×10^7^	2×10^7^	3×10^7^	2.70×10^7^±7688375	88.90±25.62
Alu2	Low range	2×10^4^	2×10^4^	4×10^4^	3.90×10^4^±9387	98.50±23.52
	High range	1×10^7^	2×10^7^	3×10^7^	3.40×10^7^±3480102	114.01±11.53
Alu3	Low range	2×10^4^	2×10^4^	4×10^4^	4.10×10^4^±4372	103.33±10.93
	High range	1×10^7^	2×10^7^	3×10^7^	2.90×10^7^±2728451	99.00±9.19
Alu4	Low range	2×10^4^	2×10^4^	4×10^4^	3.80×10^4^±4910	96.80±12.38
	High range	1×10^7^	2×10^7^	3×10^7^	3.10×10^7^±2645751	103.56±8.91
Alu5	Low range	2×10^4^	2×10^4^	4×10^4^	4.10×10^4^±3215	102.50±8.03
	High range	1×10^7^	2×10^7^	3×10^7^	2.40×10^7^±3844188	82.33±12.92

**Table V:** Prognostic ability of serum Alu for detecting cfDNA in patients with MI.

Test result variable(s)	Area	SEM	p-value	95% CI		Cut-off value	Sensitivity (%)	Specifity (%)

				Lower bound	Upper bound			
cTnI	0.903	0.029	p<0.001	0.846	0.959	1.35 (ng/ml)	80.70	84.40
CK-MB	0.752	0.049	p<0.001	0.655	0.848	28.50 (IU/l)	80.70	68.90
LDH	0.801	0.043	p<0.001	0.717	0.886	281.00 (IU/l)	70.20	82.20
Alu1	0.887	0.033	p<0.001	0.822	0.953	3.71 (log copies/ml)	77.20	95.60
Alu2	0.758	0.049	p<0.001	0.662	0.854	1.93 (log copies/ml)	100.00	48.95
Alu3	0.857	0.036	p<0.001	0.787	0.927	0.22 (log copies/ml)	100.00	60.00
Alu4	0.940	0.026	p<0.001	0.889	0.990	3.73 (log copies/ml)	86.80	95.60
Alu5	0.968	0.017	p<0.001	0.934	1.002	6.13 (log copies/ml)	86.80	100.00
Alu	0.933	0.031	p<0.001	0.871	0.994	6.40 (log copies/ml)	92.10	95.60

cfDNA, cell-free DNA; MI, myocardial infarction; CI, confidence interval; SEM, standard error of the mean; cTnI, cardiac troponin I; CK-MB, creatine kinase MB isoenzyme; LDH, lactate dehydrogenase.
